# Application of 5-Fluorouracil in management of glandular odontogenic cyst

**DOI:** 10.4317/jced.60092

**Published:** 2023-06-01

**Authors:** Fadi Titinchi, Junaid Thompson, Sanjay Ranchod

**Affiliations:** 1 Department of Maxillo-Facial and Oral Surgery, Faculty of Dentistry and WHO collaborating centre, University of the Western Cape, Cape Town, South Africa

## Abstract

Glandular odontogenic cyst (GOC) is a rare, aggressive odontogenic lesion that presents in the jaws. It is a diagnostically challenging entity due to its ability to mimic intraosseous mucoepidermoid carcinoma, botryoid cyst, surgical ciliated cyst, and radicular cyst. Treatment ranges from conservative to aggressive surgical interventions due to its varied clinical and radiological appearance and also its potential for recurrence. Aggressive surgical interventions result in the need for surgical site reconstruction, thus increasing patient morbidity. We report a case of GOC in the anterior mandible that was conservatively treated by the application of 5-Fluorouracil (5-FU). Topical application of 5-FU was selected in the management of this lesion as it has shown to be effective in reducing recurrence rates of other aggressive odontogenic lesions such as odontogenic keratocysts. To our knowledge, this is the first case reported in the literature that was successfully treated by the combination of cyst enucleation, curettage, peripheral ostectomy and application of 5-FU. No recurrence was detected after 14-month follow-up.

** Key words:**Odontogenic cysts, Fluorouracil, Recurrence.

## Introduction

Glandular odontogenic cyst (GOC) is a rare, aggressive odontogenic cyst with a recurrence rate as high as 21.6% (1). 5-Fluorouracil (5-FU) is a chemotherapeutic, anti-cancer agent administered in either intravenous or topical forms. The topical form has been successfully used in the management of basal cell carcinomas of the skin and more recently has been effective in reducing recurrences of odontogenic keratocysts (OKC) (2). To our knowledge, no previous study has evaluated the efficacy of 5-FU in the management of GOC. We describe, to the best of our knowledge, the first case of GOC that was successfully treated by enucleation, curettage and the application of 5-FU.

## Case Report

**Figure 1:**
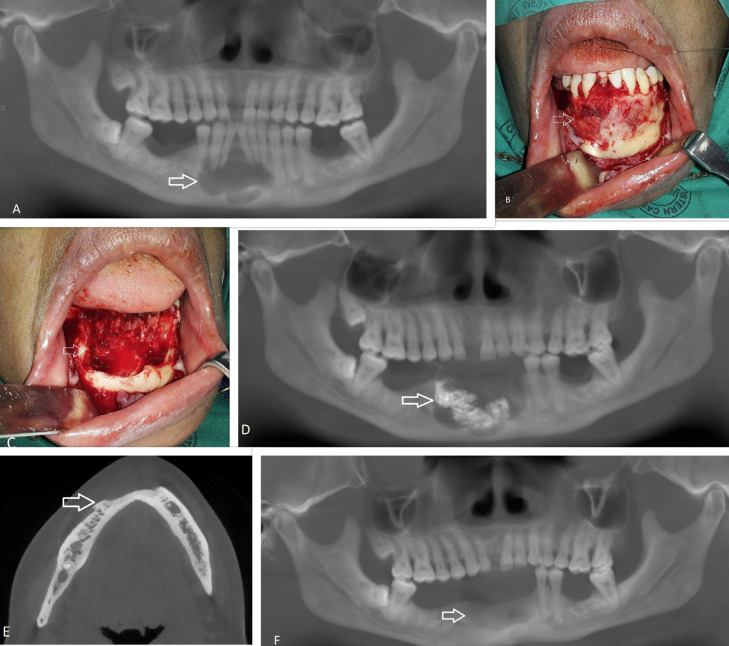
A: Panorex showing multilocular Glandular Odontogenic Cyst (GOC) in anterior mandible. B: Intra-operative image of lesion. C: Defect post enucleation of lesion. D: Day one post-operative panorex with 5-Fluoruracil soak gauze in the surgical defect. E: Axial CBCT 14 months post treatment showing new bone deposition and no sign of recurrence. F: Panorex 14 months post treatment with good bone filling in defect.

A 51-year-old virally suppressed HIV-positive female presented with a painless mass in the anterior mandible that was incidentally identified during routine radiographic examination by her dentist. Clinically, the patient had mild buccal bony expansion in the anterior mandible. Cone beam computerized tomography (CBCT) scan revealed a well circumscribed, multilocular radiolucency in the anterior mandible that extended from the first left premolar to the right canine (Fig. 1A). Subsequently, an incisional biopsy under local anaesthesia was performed to establish the diagnosis.

GOC was histologically confirmed and treatment under general anaesthesia included enucleation, curettage, peripheral ostectomy and the application of 5-FU (Fig. 1B,C). The entire cyst lining was enucleated and curetted. In addition, teeth that was associated with the cyst was extracted. Following enucleation and curettage, methylene blue was applied to the residual bony cavity and washed with saline. Peripheral ostectomy was performed to remove any residual cyst lining that stained blue. A ribbon gauze soaked in 5% 5-FU topical cream was placed into the residual bony cavity (Fig. 1D). This was subsequently removed 24 hours post-operatively. A follow-up CBCT scan was performed 14 months post-operatively which displayed good bone healing without any radiological signs of recurrence (Fig. 1E,F). The patient has been scheduled for annual follow-up to assess for recurrence. An implant-supported prosthesis has been planned for the replacement of extracted teeth.

## Discussion

GOC is a rare lesion that was recognized by the World Health Organisation (WHO) as a cyst of odontogenic origin in 1992 (3). Preceding this, it was termed a sialo-odontogenic cyst in 1987 by Padayachee and Van Wyk, who reported the first two cases (4). Later, the cyst was described to be a distinctive entity by Gardner *et al*. (1988) and subsequently the term glandular odontogenic cyst was adopted (5).

GOCs are exceedingly rare and literature reports that it makes up approximately 0.2% of all odontogenic cysts (6). GOCs are aggressive lesions that have a high rate of recurrence. A systematic review by Chranovic and Gomes analysed 169 cases in the literature and found a recurrence rate of 21.6% (1). This is less than previously thought, however, still presents a significant challenge in the management of GOC.

Histologically, GOC has similar features to both benign and malignant neoplasms of the jaws. The lesion can mimic mucoepidermoid carcinoma, botryoid cyst, surgical ciliated cyst, radicular cyst or dentigerous cyst6. Clinically, they present as slow growing, asymptomatic swellings of the jaw and less frequently present with pain and paraesthesia. GOC’s occur more frequently in the mandible (70%) than the maxilla (30%). It is most commonly found in the anterior regions of the jaws with no gender predilection (1). The mean age of diagnosis is 51 years (7).

Surgical management of GOC includes curettage, enucleation, marginal and segmental resection (1). Enucleation is the most common treatment modality but also results in the highest recurrence rate (16/63; RR= 25.4%). Curettage was associated with the second highest recurrence rate (RR= 16%). More conservative surgical methods are associated with higher rates of recurrence indicating the aggressive nature of GOC and the need for more aggressive surgical approaches. Marginal and segmental resection had recurrence rates of 12.5% and 0% respectively but lead to significant morbidity (1). Due to the high recurrence rate associated with enucleation alone, adjuvant methods (Carnoy’s solution, cryotherapy and peripheral ostectomy) should be implemented to reduce this risk in similar fashion to other aggressive odontogenic lesions. 5-FU has recently been utilized as adjuvant material for the management of aggressive odontogenic cysts.

5-FU is a chemotherapeutic agent that has been in use since 1957 for the management of colonic and breast cancers as well as skin cancers in the head and neck region. It functions by inducing cytotoxicity via reducing the production of deoxythymidine mono-phosphate which is a key intracellular molecule required for DNA repair and replication (8).

The advantages of 5-FU have been well documented in the management of OKCs. A recent study demonstrated that the use of 5-FU is superior to Carnoy’s solution (2). The study showed no recurrences in a sample of 34 patients managed with 5-FU compared to 9 recurrences in a sample of 36 patients that were treated with Carnoy’s solution (2).

The benefits of 5-FU in the management of aggressive odontogenic cysts are well established, however there are no recorded cases of GOC being managed with 5-FU as an adjuvant treatment option. This report documents the first case of GOC being successfully treated with 5-FU as an adjuvant medicament with no recurrence after 14-month follow-up. In conclusion, this report demonstrates that GOC, in addition to enucleation, curettage and peripheral ostectomy, can be successfully managed by the application of 5-FU. This conservative method of treatment may result in reduced recurrence rates without significant morbidity to the patient.
